# Accelerometer-measured physical activity and sedentary behavior in nonagenarians: Associations with self-reported physical activity, anthropometric, sociodemographic, health and cognitive characteristics

**DOI:** 10.1371/journal.pone.0294817

**Published:** 2023-12-06

**Authors:** Sari Aaltonen, Mia Urjansson, Anni Varjonen, Henri Vähä-Ypyä, Paula Iso-Markku, Sara Kaartinen, Tommi Vasankari, Urho M. Kujala, Karri Silventoinen, Jaakko Kaprio, Eero Vuoksimaa

**Affiliations:** 1 Institute for Molecular Medicine Finland FIMM, University of Helsinki, Helsinki, Finland; 2 UKK Institute for Health Promotion Research, Tampere, Finland; 3 HUS Diagnostic Center, Clinical Physiology and Nuclear Medicine, University of Helsinki and Helsinki University Hospital, Helsinki, Finland; 4 Department of Public Health, University of Helsinki, Helsinki, Finland; 5 Department of Physical Medicine and Rehabilitation, HUS Hyvinkää Hospital, Hyvinkää, Finland; 6 Faculty of Medicine and Health Technology, Tampere University, Tampere, Finland; 7 Faculty of Sport and Health Sciences, University of Jyväskylä, Jyväskylä, Finland; 8 Faculty of Social Sciences, University of Helsinki, Helsinki, Finland; Tokat Gaziosmanpasa University Tasliciftlik Campus: Tokat Gaziosmanpasa Universitesi, TURKEY

## Abstract

**Background:**

Research on device-based physical activity in the oldest-old adults is scarce. We examined accelerometer-measured physical activity and sedentary behavior in nonagenarians. We also investigated how the accelerometer characteristics associate with nonagenarians’ self-reported physical activity, anthropometric, sociodemographic, health and cognitive characteristics.

**Methods:**

Nonagenarians from a population-based cohort study (N = 38, mean age 91.2) used accelerometers during the waking hours for seven days. They also participated in a health survey and cognitive telephone interview. The Wald test and Pearson and polyserial correlations were used to analyze the data.

**Results:**

The participants’ average day consisted of 2931 steps, 11 minutes of moderate-to-vigorous physical activity and 13.6 hours of sedentary time. Physical activity bouts less than 3 minutes per day and sedentary time bouts of 20–60 minutes per day were the most common. No sex differences were found. Many accelerometer-measured and self-reported physical activity characteristics correlated positively (correlations ≥0.34, p-values <0.05). The low levels of many accelerometer-measured physical activity characteristics associated with low education (correlations ≥0.25, p-values <0.05), dizziness (correlations ≤-0.42, p-values <0.01) and fear of falling (correlations ≤-0.45, p-values <0.01). Fear of falling was also associated with accelerometer-measured sedentary behavior characteristics (correlations -0.42 or ≥0.43).

**Conclusions:**

Nonagenarians were mostly sedentary and low in physical activity, but individual variability existed. Accelerometer-measured and self-reported physical activity had a good consistency. Education, dizziness and fear of falling were consistently related to accelerometer-measured characteristics in nonagenarians.

## 1 Introduction

Nonagenarians are the fastest growing population segment in many countries [1]. Along with physical inactivity, nonagenarians’ health and high risk of cognitive impairment will be major challenges in the future decades. These issues highlight the imperative to better understand not only the physical activity behavior and the factors affecting this behavior in the oldest-old age, but also the associations between physical activity, health and cognition.

The state-of-the-art supports the use of devices in physical activity studies because devices eliminate many of the limitations associated with self-reports [2]. Most devices are simple to use, and specific intensity components can be easily yielded by using devices. However, only a few device-based physical activity studies have included nonagenarians [3] or focused exclusively on participants aged 90 and over [4–7]. Previous hip- and thigh-worn accelerometer studies have shown 98 minutes of total physical activity a day in Spanish nonagenarians (the exact time for specific intensities were not given by the authors) [4], and 50 and 13 minutes of moderate-to-vigorous physical activity a day in relatively healthy American nonagenarians [5] and Swedish nonagenarians living at home or in nursing homes [3], respectively. When armbands were used, the percentage of moderate-to-vigorous physical activity was 43% and 46% per day in a selected group of Sardinian nonagenarian men and women, respectively (the exact time was not given by the authors) [7].

The results of number of daily steps in nonagenarians have been varying as well. The Swedish study reported 4818 steps per day for nonagenarian men and women together [3], while the study of the long-living population of Sardinian nonagenarian indicated 12110 steps for men and 12799 for women [7] and the study of Italian nonagenarian population from the Mugello area 658 steps for men and 883 for women [6]. In terms of sedentary time, 552 minutes was monitored by a thigh-worn accelerometer among those Swedish nonagenarians living at home or in nursing homes [3], which was consistent with 597 minutes of sedentary time monitored by a hip-worn accelerometer among American nonagenarians living in retirement communities [8].

Given only few studies, some of which had highly selected samples, there is the need for more research that focuses on device-based physical activity in nonagenarians. The further need is justified by the fact that only one previous study has focused on the association between device-based and self-reported physical activity, indicating no significant association between these factors [3]. Finally, very little is known about the associations of device-measured characteristics with health and cognition in nonagenarians. The Italian Mugello Study [6] that suggested the low number of daily steps for nonagenarians is, to our knowledge, the only study that has also focused on this association, showing that the number of daily steps correlated significantly with nonagenarians’ cognitive (positively) and depression status (negatively).

In the present study, we have taken many of the previous issues into account and provide novel information. By using a population-based sample, we investigated nonagenarians’ accelerometer-measured physical activity and sedentary behavior and their correspondence with nonagenarians’ own ratings of their physical activity behavior. We also examined anthropometric, sociodemographic, health and cognitive correlates of accelerometer-measured physical activity and sedentary behavior. We expected substantial individual differences but no sex differences in accelerometer-measured physical activity and sedentary behavior characteristics in nonagenarians. Additionally, we hypothesized that the accelerometer-measured and self-reported physical activity characteristics correlate at least moderately.

## 2 Methods

**Fig 1:**
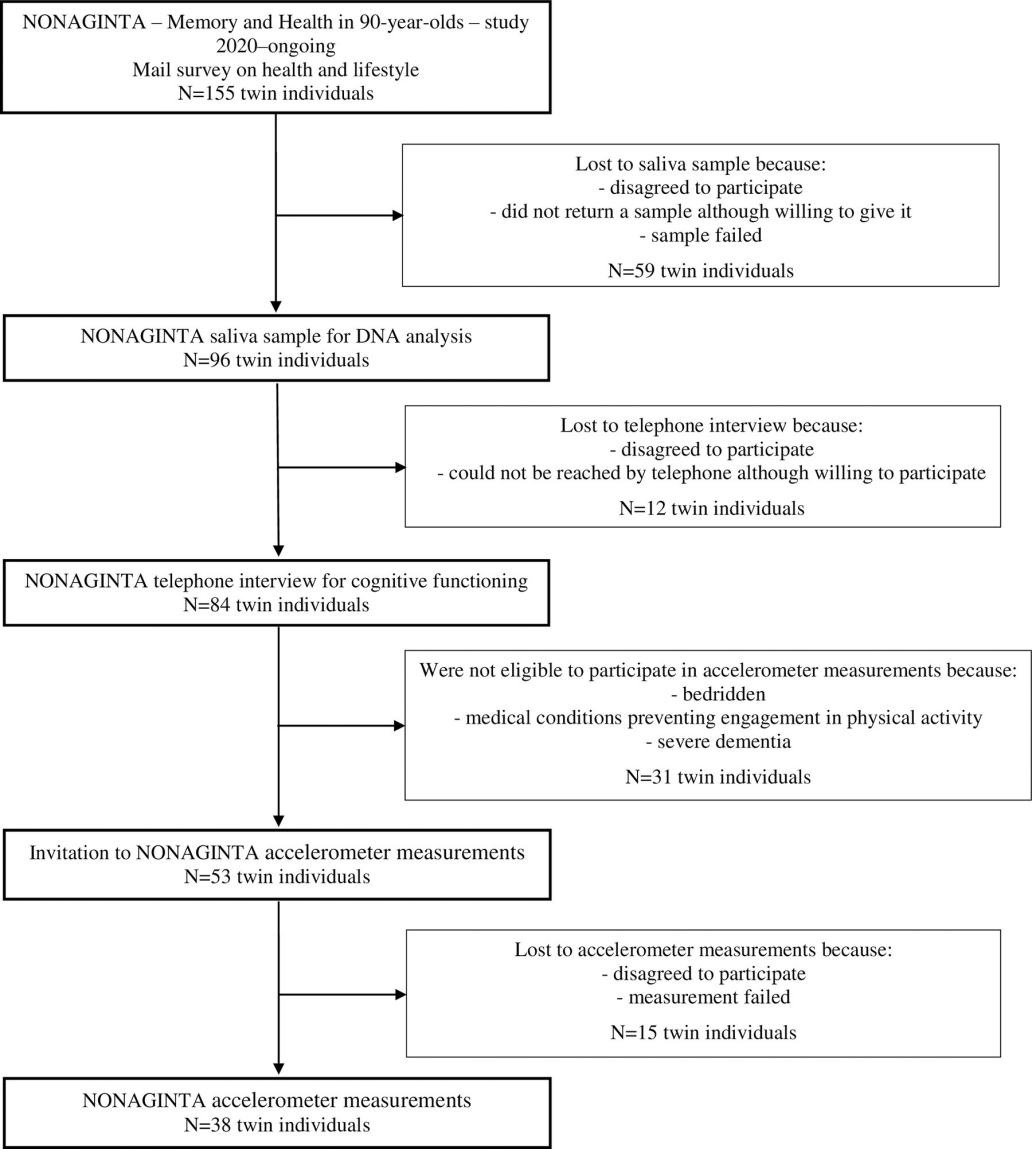
Flow chart outlining the protocol and number of participants in the NONAGINTA–Memory and Health in 90-year-olds–study.

Participants were from the ongoing NONAGINTA–Memory and Health in 90-year-olds–study that is aimed to investigate 90-year-old twins from the population-based older Finnish Twin Cohort study [9]. The data collection of the NONAGINTA study was launched in 2020 (Fig 1). The data have been collected by 1) a mail survey on health and lifestyle factors, including physical activity, 2) a saliva sample for DNA analysis and 3) a telephone interview for cognitive functioning. In addition, 4) accelerometer measurements on daily physical activity and sedentary behavior were available for a sub-sample of those who participated in the telephone interview. Chronic diseases or cognitive disorders, such as mild dementia, were not automatically considered as exclusion criteria for data collection given multiple morbidities and the high prevalence of dementia in nonagenarians [10, 11]. However, those participants who were bedridden, had medical conditions preventing their engagement in any physical activity or were considered not to be able to participate in device-based assessments due to cognitive impairment were excluded from the accelerometer data collection. Individuals with reduced mobility were included in the accelerometer data collection regardless of using walking aids or needing help from a caregiver. The response rate of the mail survey was 28%. Of those, 54% participated in the telephone interview, and the participation rate of accelerometer data collection was 72% (of those who were eligible to participate). Altogether, we had questionnaire, interview and accelerometer data on 34–38 nonagenarian participants, depending on the measure (50% men, median age 91, age range 90–98 years). There were four complete twin pairs in our dataset (both co-twins of a pair participated).

### 2.1 Questionnaire-based variables

#### 2.1.1 Physical activity

The questionnaire-based assessment of physical activity behavior was based on the structured and validated questions, which were also used in the previous surveys of the older Finnish Twin Cohort study [12, 13]. The participants reported their overall amount of year-round physical activity as well as their monthly frequency, duration, and intensity of physical activity sessions. The exact questions were: “How do you describe the amount of your year-round physical activity?”, “How often do you exercise or be physically active in a month?”, “How long does one session of the physical activity last on average?”, and “Is your physical activity about as tiring on average as:”. The response options for the questions are shown in the S1 File. Based on these structured questions, we constructed daily metabolic equivalents (METs) expended per hour to represent overall physical activity behavior. The calculation formula of MET hours per day was the following: physical activity frequency × physical activity duration × physical activity intensity. The MET values for physical activity intensity were: 4 for intensity corresponding to walking, 6 for intensity corresponding to vigorous walking to jogging, 10 for intensity corresponding to jogging, and 13 for the intensity corresponding to running.

#### 2.1.2 Education

The self-reported highest educational degree achieved was the measure of education. The level of education was classified into eight groups: 1) less than compulsory education, 2) compulsory education (six years in our participants), 3) compulsory education and at least one-year vocational training, 4) lower secondary education, 5) lower secondary education and at least one-year vocational training (or upper secondary studies), 6) upper secondary education, 7) upper secondary education and at least one-year vocational training (or tertiary education studies), and 8) tertiary education (university or polytechnic college).

#### 2.1.3 Anthropometric and health-related variables

The height and weight of the participants were self-reported. Based on the self-reports, body mass index (BMI) was calculated as the ratio between weight and height in meters squared (kg/m^2^). The participants also rated how they perceived their health in general and reported whether they ever feel dizzy or off balance. The given response options for the subjective health status were: 1) very poor, 2) poor, 3) fair, and 4) good. For dizziness and poor balance, the response options were: 1) never or hardly ever, 2) sometimes, causing some harm, and 3) often, causing great harm. Fear of falling was assessed with the question “Are you afraid of falling?” with the response options of 1) never, 2) occasionally, 3) often, and 4) constantly.

#### 2.1.4 Depressive symptoms

The Center for Epidemiologic Studies–Depression (CES-D) scale was used to assess depressive symptoms [14]. The CES-D scale includes 20 items assessing the frequency of depressive symptoms during the past week. The CES-D score is the sum of 20 items, each answered from 0 to 3. Thus, the sum score ranges between 0 and 60, with higher scores indicating higher levels of depressive symptoms. We also used a validated cut-off of 20 –those with higher values were identified as participants with clinically significant levels of depressive symptoms [15]. The CES-D scale has shown good reliability and validity also among older adults [16–19].

### 2.2 Telephone-administered cognitive testing

In the NONAGINTA study, two validated telephone interviews were used: 1) telephone assessment of dementia (TELE) [20–22] and 2) the modified version of the Telephone Interview for Cognitive Status (TICS-m) (this was further modified by including three trials in the 10-word list learning task) [23]. These telephone interviews were carried out by a trained research nurse. TELE correlates significantly with the Mini Mental State Examination, with a correlation coefficient over 0.8, and can therefore be considered as a cognitive screening instrument [21].

Cognitive status was based on a validated Finnish TELE (score range 0–20 with 2 cut-off points): cognitively impaired (scores <16) and cognitively healthy (scores >17.5) [21]. We also utilized the category of mild impairment in cognitive function (scores 16–17.5), although we note that as a cognitive instrument the TELE is not a validated measure of mild cognitive impairment [24]. In addition to this categorical cognitive status, we measured episodic memory and semantic fluency: cognitive domains sensitive to aging and dementia-related changes. Episodic memory was measured with a 10-word list learning task included in the TICS-m. We used both the total number of words recalled in three trials (score range 0–30) and the number of words recalled after a short delay (score range 0–10) as measures of immediate and delayed recall, respectively. Semantic fluency was assessed by a one-minute animal naming task (i.e., the participants were asked to name as many animals as possible in one minute). The detailed description and validation of the Finnish TELE and TICS-m can be found elsewhere [21, 25].

### 2.3 Accelerometer-measured physical activity and sedentary behavior

The participants used a hip-worn tri-axial accelerometer (UKK RM42, UKK Terveyspalvelut Oy, Tampere, Finland) to monitor their daily physical activity and sedentary behavior [26]. The accelerometer has been successfully used in large population-based studies, including older adults [26, 27]. The device and instructions were mailed to the participants between April 1, 2021 and December 31, 2022. The participants were told to wear the accelerometers for seven consecutive days. During the waking hours, they were told to wear the accelerometer in an elastic waistband on the right side of the hip except during all water activities, such as bathing, taking a shower, and swimming. After seven days, the participants were asked to mail the device back for data analysis.

Data from the accelerometers were recorded at a sampling frequency of 100 Hz. The analyses of raw acceleration data are based on algorithms that employ the mean amplitude deviation (MAD) of the raw acceleration signal and the angle for posture estimation (APE) of the body [28]. These algorithms are high in validity and, together, the metrics provide about 1.2 MET accuracy for intensity estimation for bipedal locomotion over a wide range of speed and 90% accuracy in body posture in free-living conditions [28–30]. For the analyses, MAD and APE values were determined for each 6-second epoch. The epoch-wise MAD values were expressed in METs. The epoch-wise MET values were smoothed by calculating an exponential moving average for each epoch time point.

We pre-specified to report our nonagenarians’ daily step count and time spent in physical activity and sedentary behavior, including time spent in moderate-to-vigorous physical activity. Compared to younger adults, the impulse required to detect a step was set at a lower level (i.e., 0.02 gravity seconds instead of 0.03 gravity seconds) among these nonagenarian participants because of the common finding of a shortened step length in older adults [31]. Physical activity was categorized as time spent in sustained physical activity bouts lasting 1) less than 3 minutes, 2) from 3 to 10 minutes, and 3) more than 10 minutes. In the results section, we report the accumulated time of these physical activity bouts. Mean daily moderate-to-vigorous physical activity time was defined as physical activity of at least 3 METs. Sedentary behavior was measured as overall sedentary time per day and was further categorized as time spent in sustained sedentary time bouts lasting 1) less than 20 minutes, 2) from 20 to 60 minutes, and 3) more than 60 minutes. Similar to physical activity bouts, we report the accumulated time of these sedentary bouts in the results section. Mean daily sedentary time was defined as under 1.5 METs during lying down, sitting, or standing.

Based on a consensus of the time criterion for adequate accelerometer data collection [32], we followed the principle that at least 10 hours wear-time a day was needed to be met to be included in the analyses–non-wear time was defined as a sum of at least 120 minutes of consecutive zero acceleration. To minimize intra-individual variation, at least a 4-day monitoring period was also required to be included in the analyses.

### 2.4 Statistical analyses

We reported questionnaire- and interview-based physical activity, anthropometric, sociodemographic, health, and cognitive characteristics as well as accelerometer-measured physical activity and sedentary behavior characteristics for men and women. Next, we used the Wald test to assess whether the characteristics differed significantly between sexes. The Wald test was also used to investigate whether selection bias occurred in accelerometer data collection. Regarding selection bias, we assessed whether questionnaire-based characteristics and cognitive functioning measured by telephone interview differed significantly between those who did and did not participate in the accelerometer data collection. Because there were four complete twin pairs in our dataset, we controlled for family structure by including the pair number as a clustering variable. Thus, standard errors and p-values were adjusted for the non-independency of twin data.

To see whether self-reported physical activity, anthropometric, sociodemographic, health, and cognitive characteristics were associated with accelerometer-measured physical activity and sedentary behavior characteristics, we calculated correlations between these factors. We used Pearson correlation between continuous variables (e.g., BMI and accelerometer-measured characteristics), whereas polyserial correlations were used to estimate the associations between ordinal and continuous variables (e.g., subjective health status and accelerometer-measured characteristics). In all analyses, Stata 16.1 software (StataCorp, College Station, Texas, USA) was used, and the threshold for the statistically significant p-value was set at p<0.05.

### 2.5 Ethics

The Ethics Committee of the Hospital District of Helsinki and Uusimaa approved the NONAGINTA study protocol on May 8, 2020 (mail survey, telephone interview and DNA sample) and December 16, 2020 (accelerometer data collection). All study methods were carried out in accordance with the approved guidelines and the Helsinki Declaration. All participants provided written informed consents for the main study (survey, saliva sample and telephone interview) and for accelerometer measurements.

## 3 Results

Table 1 and S1 Table illustrate self-reported physical activity, anthropometric, sociodemographic, health and cognitive characteristics of the nonagenarian participants. Men were taller and weighed more than women but no significant differences were observed in BMI (25.9 kg/m^2^ *versus* 24.4 kg/m^2^, p = 0.21). Most of the participants had a compulsory education only and they reported their health to be at least fair and having no dizziness or constant fear of falling. Men and women did not differ in immediate recall (men mean (M) = 12.2, women M = 12.6, p = 0.71), delayed recall (men M = 1.8 and women M = 2.6, p = 0.28), or semantic fluency performance (men M = 17.4 and women M = 16.9, p = 0.67). Based on the TELE, 6 of 37 participants (16%) were cognitively impaired and 14 of 37 participants (38%) had mild impairment in cognition. Altogether, 4 of 38 participants (11%) reported a clinically significant number of depressive symptoms. As shown in Table 1, men reported to be significantly more often physically active than women (p = 0.03). However, the level of physical activity expressed as MET hours/day was the same for men and women (2.3 MET hours/day *versus* 1.5 MET hours/day, p = 0.10). The majority of participants reported that the intensity of their physical activity sessions was as tiring as walking (the MET value of 4 corresponds to walking).

**Table 1:** Demographics and cognitive, health, and physical activity characteristics in nonagenarian men (n = 19) and women (n = 19).

Characteristics	Men	Women	
	Mean (*SD*)	Mean (*SD*)	p-value
**Age** (years)	91.0 (1.3)	91.5 (1.9)	0.42
**Height** (cm)	172.7 (5.5)	155.8 (4.9)	<0.001
**Weight** (kg)	72.7 (9.2)	62.8 (10.0)	0.01
**BMI** (kg/m^2^)	24.4 (2.9)	25.9 (4.1)	0.21
**Education**			0.91
≤ Lower secondary education	12	10	
> Lower secondary education	7	9	
**Subjective health status**			0.19
≤ Poor	1	3	
Fair	12	13	
Good	6	3	
**Dizziness/poor balance**			0.80
≤ Hardly ever	10	11	
≥ Sometimes	9	8	
**Fear of falling**			0.33
≤ Occasionally	16	16	
≥ Often	3	3	
**Words recalled in three trials**	12.2 (3.6)^a^	12.6 (3.9)	0.71
**Delayed word list recall**	1.8 (1.7)^a^	2.6 (2.4)	0.28
**Semantic fluency**	17.4 (4.4)^a^	16.9 (3.5)	0.67
**TELE category**			0.56
Cognitive impairment	4^a^	2	
Mild impairment	6^a^	8	
Cognitively healthy	8^a^	9	
**Depressive symptoms** *	13.2 (4.5)^a^	15.3 (7.1)^a^	0.27
**Clinically significant depressive symptoms**			0.30
Yes	3	1	
No	16	18	
**Amount of physical activity**			0.27
≤ Little	4	6	
Moderate	10	9	
≥ Quite a lot	5	4	
**Physical activity frequency**			0.03
≤ 3–5 times a month	3	4^b^	
6–10 times a month	1	6^b^	
11–19 times a month	4	3^b^	
More than 20 times a month	11	3^b^	
**Physical activity duration**			0.85
< 30 min	5	4^b^	
30 min to < 1 hour	9	7^b^	
≥ 1 hour	5	5^b^	
**Physical activity intensity**			0.20
Walking	14	15^c^	
Alternatively walking and jogging	4	2^c^	
≥ Jogging	1	0^c^	
**MET hours/day**	2.3 (1.7)	1.5 (1.3)^b^	0.10

*Notes*: SD = standard deviation; BMI = body mass index; TELE = Telephone assessment for dementia; MET = metabolic equivalent

* Sum score ranging 0–60, higher values indicate depressive symptoms

^a^ = the number of participants is 18

^b^ = the number of participants is 16

^c^ = the number of participants is 17.

### 3.1 Accelerometer-measured physical activity and sedentary behavior

All participants wore the accelerometer for at least four days and for at least 10 hours a day. The mean time the participants carried the devices a day was 16:23:00 h:min:sec (men 15:54:44 and women 16:51:17). On average, the participants took 2931 steps a day during the measurement week: men 2924 (range 518–9878) and women 2936 (range 528–11190) steps. The average time of total physical activity was 111.4 minutes (men 116.0 and women 106.9). Out of this total physical activity time, 100.7 minutes (men 104.3 and women 97.1) were spent in light and 10.7 minutes (men 11.6 and women 9.8) in moderate-to-vigorous physical activity in a day. Physical activity bouts shorter than 3 minutes were the most often recorded amount of physical activity (Table 2). The mean sedentary time per day was 13:35:26 h:min:sec (men 12:57:58 and women 14:12:54). Uninterrupted sedentary time bouts that lasted from 20 to 60 minutes were the most common for both men and women. Table 2 illustrates that neither accelerometer wear time nor any of the accelerometer-measured physical activity and sedentary behavior characteristics differed between sexes (all p-values >0.19).

**Table 2:** Accelerometer wear time and accelerometer-measured physical activity and sedentary behavior in nonagenarian men (n = 19) and women (n = 19).

Characteristics	Men	Women	
	Mean (*SD*)^†^	Mean (*SD*)^†^	p-value
Wear time per day (h:min:sec)	15:54:44 (2:06:14)	16:51:17 (2:08:35)	0.20
Steps per day (number)	2924 (2473)	2936 (2422)	0.99
Total PA per day (min)	116.0 (62.0)	106.9 (57.5)	0.65
Light PA per day (min)	104.3 (48.5)	97.1 (47.2)	0.66
MVPA per day (min)	11.6 (17.2)	9.8 (13.2)	0.70
<3-min bouts of PA per day (min)*	78.5 (37.6)	73.9 (33.7)	0.69
3–10-min bouts of PA per day (min)*	16.4 (8.6)	14.5 (9.8)	0.50
>10-min bouts of PA per day (min)*	21.0 (25.4)	18.5 (24.4)	0.77
ST per day (h:min:sec)	12:57:58 (2:40:32)	14:12:54 (2:58:59)	0.48
<20-min bouts of ST per day (min)	263.4 (104.8)	282.9 (105.6)	0.56
20–60-min bouts of ST per day (min)	281.8 (116.2)	322.2 (83.9)	0.25
>60-min bouts of ST per day (min)	278.6 (207.1)	301.1 (168.3)	0.72

*Notes*: min = minute; MVPA = moderate-to-vigorous physical activity; PA = physical activity; ST = sedentary time; SD = standard deviation; h = hours; min = minutes; sec = seconds

^†^ Except where indicated other

* All physical activity intensities are included.

### 3.2 Correlates of accelerometer-measured characteristics

We found that self-reported physical activity characteristics, except the intensity of physical activity, correlated significantly with the accelerometer-measured number of steps, moderate-to-vigorous physical activity and bouts of physical activity lasting more than 10 minutes (Table 3). The participants’ overall physical activity behavior expressed as MET hours/day also correlated significantly with the accelerometer-measured total and light physical activity as well as with the accelerometer-measured bouts of physical activity less than 3 minutes. In addition, the participants’ self-reported frequency and intensity of physical activity correlated significantly with the accelerometer-measured total physical activity (correlation (r) = 0.35) and bouts of physical activity less than 3 minutes (r = 0.38), respectively.

**Table 3:** Correlations between questionnaire-based and accelerometer-measured physical activity variables in nonagenarians (n = 38).

	Accelerometer-measured						
**Questionnaire-based**	Number of steps	Total PA	Light PA	MVPA	< 3-min bouts of PA	3–10-min bouts of PA	>10-min bouts of PA
Amount of PA	0.34*	0.32	0.32	0.25	0.22	0.32	0.35**
PA frequency (*n* = 35)	0.44***	0.35*	0.28	0.50***	0.23	0.18	0.48***
PA duration (*n* = 35)	0.45***	0.29	0.22	0.45***	0.20	0.06	0.42***
PA intensity (*n* = 36)	0.14	0.30	0.36	-0.01	0.38*	0.30	0.08
MET hours/day (*n* = 35)	0.60***	0.50**	0.41*	0.65***	0.34*	0.27	0.61***

*Notes*: min = minute; MVPA = moderate-to-vigorous physical activity; PA = physical activity. Pearson correlations were reported for the MET variable. Polyserial correlations were reported for the amount, frequency, duration and intensity of physical activity. Statistically significant results are in bold font

* = p<0.05

** = p<0.01

*** = p<0.001.

Our next analyses concerned the associations of accelerometer-measured physical activity and sedentary behavior characteristics with self-reported anthropometric, sociodemographic, health, and cognitive characteristics (Table 4). The results revealed that education was positively correlated with the daily number of steps (r = 0.25), total physical activity (r = 0.27), light physical activity (r = 0.27), and the bouts of physical activity less than 3 minutes (r = 0.28). Compared to those with poor subjective health and higher BMI, those with good subjective health and lower BMI spent less sedentary time per day (r = -0.45 and r = 0.35, respectively). The high feelings of both dizziness and fear of falling were strongly and consistently associated with the lower values of accelerometer-measured physical activity characteristics (correlations (rs) -0.68 to -0.42). Fear of falling was also associated with accelerometer-measured sedentary behavior characteristics, but the directions of these associations were less consistent than those of accelerometer-measured physical activity (rs -0.42 or ≥0.43). Episodic memory, semantic fluency, cognitive status, or depressive symptoms were not related to any of the accelerometer-measured characteristics, although many of the correlations between clinically significant depressive symptoms and accelerometer characteristics were reasonable high (rs ≤-0.25), as shown in Table 4. We further compared the accelerometer-measured characteristics between those who had and had not clinically significant depressive symptoms, but found no statistically significant differences (all p-values >0.09).

**Table 4:** Pearson and polyserial correlations between self-reported questionnaire- and interview-based anthropometric, sociodemographic, health, and cognitive variables and accelerometer-measured physical activity and sedentary behavior variables (means per day) in nonagenarians (n = 38).

	Accelerometer-measured										
**Questionnaire- and interview-based**	Number of steps	Total PA	Light PA	MVPA	<3-min bouts of PA	3–10-min bouts of PA	>10-min bouts of PA	ST	<20-min bouts of ST	20–60-min bouts of ST	>60-min bouts of ST
BMI	-0.17	-0.14	-0.09	-0.27	-0.05	-0.13	-0.22	0.35*	0.03	0.16	-0.03
Education	0.25*	0.27*	0.27*	0.20	0.28*	0.08	0.22	-0.28	-0.08	-0.24	0.10
Subjective health status	0.27	0.21	0.23	0.09	0.20	0.16	0.16	-0.45***	0.08	-0.20	-0.15
Dizziness/poor balance	-0.61***	-0.47***	-0.42**	-0.61***	-0.19	-0.20	-0.56***	0.15	-0.24	-0.03	0.17
Fear of falling	-0.68***	-0.52***	-0.45**	-0.65***	-0.50***	-0.12	-0.52***	0.47**	-0.42**	-0.04	0.43*
Words recalled in three trials (*n* = 37)	0.13	0.11	0.12	0.05	0.09	-0.02	0.13	0.08	0.02	0.10	-0.03
Delayed word list recall (*n* = 37)	0.08	0.01	-0.01	0.04	-0.11	-0.03	0.19	0.06	-0.22	-0.16	0.14
Semantic fluency (*n* = 37)	-0.09	-0.13	-0.15	-0.05	-0.15	-0.21	-0.03	-0.26	-0.15	-0.23	0.01
TELE category (*n* = 37)	0.07	0.07	0.07	0.05	0.12	-0.16	0.06	0.01	0.01	-0.14	0.05
Depressive symptoms (*n* = 36)	-0.03	0.05	0.07	0.00	0.05	0.15	0.00	0.20	0.00	-0.03	0.08
Clinically significant depressive symptoms	-0.40	-0.18	-0.15	-0.26	-0.25	0.09	-0.09	-0.06	-0.30	-0.30	0.22

*Notes*: min = minute; MVPA = moderate-to-vigorous physical activity; PA = physical activity; ST = sedentary time; BMI = body mass index; TELE = Telephone assessment for dementia (1 = cognitive impairment, 2 = mild impairment, 3 = cognitively healthy). Pearson correlations were reported for BMI, words recalled in three trials, semantic fluency and depressive symptoms. Polyserial correlations were reported for education, subjective health status, dizziness/poor balance, fear of falling, delayed word list recall, TELE category and clinically significant depressive symptoms. Statistically significant results are in bold font

* = p<0.05

** = p<0.01

*** = p<0.001.

### 3.3 Selection bias analyses

Those who participated in the accelerometer data collection (N = 38) reported higher education (difference -1.15, 95% CI -2.16 to -0.13), higher health status (difference -0.24, 95% CI -0.47 to -0.01), felt significantly less dizzy or off balance (difference 0.42, 95% CI 0.17 to 0.67), and had less fear of falling (difference 0.61, 95% CI 0.29 to 0.94) than those who participated in the health survey but not in the accelerometer data collection (N = 117). Those wearing accelerometers also recalled more words immediately (difference -1.75, 95% CI -3.47 to -0.03), had better semantic fluency (difference -3.08, 95% CI -5.05 to -1.11), were more likely to be cognitively healthy (difference -0.38, 95% CI -0.75 to -0.02) and had less clinically significant levels of depressive symptoms (difference 0.18, 95% CI 0.05 to 0.31) than those who did not to participate in the accelerometer data collection. They also reported higher overall amount, duration, and intensity of physical activity (differences -0.49, 95% CI -0.86 to -0.12; -0.41, 95% CI -0.78 to -0.04 and -0.19, 95% CI -0.34 to -0.04, respectively) compared to those not wearing accelerometers.

## 4 Discussion

This study was set to examine accelerometer-measured physical activity and sedentary behavior in Finnish nonagenarians derived from a population-based sample. We investigated how the accelerometer characteristics associate with nonagenarians’ self-reported physical activity, anthropometric, sociodemographic, health, and cognitive characteristics. On an average day, the participants took 2931 steps and had 111 minutes of physical activity, of which nearly 11 minutes were moderate-to-vigorous physical activity. The average daily sedentary time was 13 and half hours. As expected, we did not observe differences in accelerometer-measured characteristics between sexes. However, in line with our hypothesis, substantial individual differences existed in all accelerometer-measured characteristics both in men and women.

In general, nonagenarians were highly sedentary and their physical activity level was low in our study. The most common physical activity and sedentary behavior characteristics recorded during the measurement week were the very short bouts of physical activity and medium-length bouts of sedentary time. Moreover, our results indicated a significant correlation between most accelerometer and self-reported physical activity items. Highly educated nonagenarians were the most active based on the time of total and light physical activity but also based on the number of steps and short bouts of physical activity. High BMI and low subjective health indicated high overall sedentary time in nonagenarians. Not surprisingly, strong feelings of dizziness and fear of falling were clearly shown to be associated with a decreased level of physical activity. Moreover, those nonagenarians who had the greatest fear of falling were overall more sedentary and spent more time in prolonged bouts of sedentary behavior.

Even though a single nonagenarian participant took a large number of steps (more than 11,000) in the present study, the average number of steps taken by nonagenarians in a day (i.e., 2931) was approximately half of the minimal number of steps a day found to be beneficial to health in adults (i.e., 7000 steps, reached by 3 participants in our study) [33]. Moreover, there is evidence that mortality is lower among those older individuals who take more than 3000 steps per day [34, 35] (13 participants in our study). The Italian nonagenarians from a Sardinian longevous population [7] took many more steps per day compared to our participants (12500 *versus* 2931), whereas Swedish nonagenarians [3] were more consistent with our participants (4818 *versus* 2931). Some of the discrepancies between these results could be attributed to differences in health status and sample characteristics but also due to devices and tracking times used. Moreover, it seems that hip- and thigh-worn devices are more consistent in their step number results (our results and Dohrn et al. 2020 [3]), while armbands have resulted in the highest [7] and lowest [6] numbers of steps found in nonagenarians. This may partly be due to the fact that the exact placement of the device on the arm also affects results [36].

Our results indicating no sex differences in any of the accelerometer-measured characteristics are in line with previous studies that found no differences in daily step count [3, 6, 7] or in daily physical activity level [6, 7] between nonagenarian men and women. However, some previous studies have reported sex differences in total energy expenditure in nonagenarians [5, 7, 37]. Out of all physical activity we recorded in nonagenarians, the time of moderate-to-vigorous physical activity was only about 11 minutes a day. This result is similar to those of Swedish nonagenarians [3] but less than those of American [5] or Italian nonagenarians [7]. In terms of sedentary behavior, the levels we observed (i.e., men 13 hours and women 14 hours) were more than those observed by the previous studies of Dohrn et al. (2020) [3] and Bellettiere et al. (2015) [8]. Variations in health criteria of study participants may partly explain the conflicting results, but the use of different cut-off points in evaluating accelerometer-measured moderate-to-vigorous physical activity in older adults have also been shown to provide conflicting estimates [38]. Moreover, the inconsistencies can partly be due to the fact that we investigated nonagenarians despite their need for walking assistance, while some previous studies (e.g., Dohrn et al. (2020) [3] and Bellettiere et al. (2015) [8]) investigated only nonagenarians who were able to move indoors without assistance.

The most interesting finding to emerge from our study was that nonagenarians’ own perception of their average physical activity behavior corresponds with a 7-day accelerometer measurement, except for the intensity of physical activity. According to our knowledge, there was only one previous study on this topic before our study [3], but that study found no association between device-based and self-reported physical activity results. Due to these conflicting results, replications are needed, but our result might imply that self-reported physical activity items (other than physical activity intensity) could be considered as indicators of actual physical activity level in nonagenarians. However, it is good to note that, in particular, if physical activity intensity is of interest, individual physical fitness levels are recommended to take into account [39, 40]. Additionally, a note of caution is due here because of the size of our sample, but still our suggestion is supported by substantial positive correlations between many accelerometer-based and self-reported estimates of physical activity in the previous large studies among adults younger than 90 years [41, 42].

Our results indicated that nonagenarians’ subjective impressions of the average intensity of their physical activity sessions do not correspond with the intensity of their accelerometer-measured physical activity. Most of our nonagenarians reported that the average intensity of their physical activity sessions was as tiring as walking. The impression the participants have of their physical activity intensity can partly be due to the fact that walking is the most common form of physical activity among older adults [43]. We used the MET values based on adult populations in our accelerometer measurements and, therefore, the cut points of MET values may have been set too high for nonagenarians. This may have caused some misclassifications [38].

Our study further showed that those with a higher education had higher physical activity levels, partly supporting previous research showing that physical activity has a positive relationship with cognitive status [6] and healthy cognitive aging [44]. However, none of the specific cognitive variables (episodic memory, semantic fluency, or cognitive status) associated with accelerometer characteristics. Not even the number of daily steps, which has previously been shown to be a positive correlate of cognition among Italian nonagenarians from the Mugello area [6]. This is in line with a previous study suggesting that lifestyle factors are not predictive of dementia in the oldest old individual [45] and also with a study indicating that physical activity interventions do not have an effect on cognition in nonagenarians [46]. However, in general, physical activity and exercise training have been shown to improve physical functioning, mobility and strength in nonagenarians [47, 48]. The Italian study of nonagenarians [6] from the Mugello area also suggested that the high number of daily steps associates negatively with depression status, but we could not find a similar association. Our results further revealed that the healthier the nonagenarians reported to be, the less time they spent in sedentary behaviors. However, this phenomenon may not be as simple as this self-reported variable of general health implies, because our results also revealed that lower levels of clinically significant depressive symptoms were having a trend toward a higher likelihood of sedentary behavior bouts lasting up to 60 minutes. This suggests that mentally healthy nonagenarians might spent more, not less, time in short- and medium-length sedentary behaviors.

A major limitation of the current study was a limited sample size, although nonagenarian accelerometer data combined with survey and interview data, such as ours, is still unique. Our sample size was small but consistent with the sample sizes of previous studies in the field [3–8]. Nevertheless, to test the robustness of our results, replications with larger datasets are needed. Meta-analyses would also be one option to provide stronger evidence. Another limitation of our study was a cross-sectional design. A longitudinal study design would have been more informative about the long-term associations and potential causality. However, a great strength of our study is that we have both device-based and self-reported physical activity data measured at the same time point. It is also worth noting that our accelerometer-measured physical activity variables are not fully comparable with our structured self-reported physical activity survey items but rather different forms of the same behavior (a 7-day accelerometer measurement *versus* the participants’ self-reports on their average physical activity behavior). Questionnaire-based physical activity behavior and the telephone interview of cognitive status used in this study are based on validated measures [12, 13, 21]. The algorithms of the accelerometer used were also high in validity [28–30].

The dataset we used was drawn from a population-based study cohort with equal sex representation and moderate response rates in the mail survey and telephone interview, which contributes to fewer selection biases. Even though we did not automatically exclude those with chronic diseases, cognitive disorders, reduced mobility or a need for walking aids, some selection bias existed with regards to accelerometer data collection. Thus, our accelerometer data should not be regarded as a representative sample of all nonagenarians. On the other hand, the consistency of our findings with the previous population-based accelerometer study of Finnish participants aged 70–85 years [49] suggests that our dataset may represent reasonably well Finnish nonagenarians. Our participants were twin individuals but studies have shown that twins do not differ from the general population in many health-related traits and behaviors or in morbidity [50–52].

A great seasonal variation in Finland with very different circumstances for outdoor activities in summer and winter is also a factor that should be considered because it strongly affects the population’s physical activity behavior. Our accelerometer data collection was conducted in the spring, summer, and fall seasons. In winter, snow and ice on the walking paths may prevent some of the nonagenarians from moving outdoors. Given that walking is the most preferred type of physical activity among older adults [43], the lack of outdoor walking would naturally result in lower levels of physical activity.

To conclude, our study provided novel information on device-based physical activity and sedentary behavior and their correlates in nonagenarians. A clinically important finding was that most of the accelerometer-measured physical activity characteristics correlated significantly with self-reported physical activity, indicating a good usability for many self-reported items in everyday settings. Our findings further suggested no sex differences in accelerometer-measured characteristics in nonagenarians, indicating that sexes behave similarly in the last decades of life. The evidence from this study indicated that there is a beneficial effect of higher education on physical activity, and that the high level of sedentary time seems to be associated with poor subjective health and high BMI still in late old age. The current data also highlight the relevance of feelings of dizziness and fear of falling as contributing factors in nonagenarian’s physical activity and sedentary behavior. However, given our limited sample size, caution is recommended when interpreting our results. Larger studies, including longitudinal aspects as well, can help in understanding the antecedents and underpinnings of both physical and mental health in this fast-growing understudied population segment.

## Supporting information

S1 File Physical activity questions used in the NONAGINTA–Memory and Health in 90-year-olds–study. (DOCX)

S1 Table Demographics and cognitive, health, and physical activity characteristics in nonagenarian men (n = 19) and women (n = 19). (DOCX)

## References

[1] He W, Goodkind D, Kowal P. An Aging World: 2015. International Population Reports.1st ed. Washington, DC, USA: U.S. Government Publishing Office; 2016.

[2] Helmerhorst HJ, Brage S, Warren J, Besson H, Ekelund U. A systematic review of reliability and objective criterion-related validity of physical activity questionnaires. Int J Behav Nutr Phys Act. 2012;9:103. doi: 10.1186/1479-5868-9-103 22938557 PMC3492158

[3] Dohrn IM, Gardiner PA, Winkler E, Welmer AK. Device-measured sedentary behavior and physical activity in older adults differ by demographic and health-related factors. Eur Rev Aging Phys Act. 2020;17(1):8. doi: 10.1186/s11556-020-00241-x 32537028 PMC7291490

[4] Hernández-Vicente A, Santos-Lozano A, Mayolas-Pi C, Rodríguez-Romo G, Pareja-Galeano H, Bustamante N, et al. Physical activity and sedentary behavior at the end of the human lifespan. J Aging Phys Act. 2019;27(6):899–905. doi: 10.1123/japa.2018-0122 31034321

[5] Johannsen DL, DeLany JP, Frisard MI, Welsch MA, Rowley CK, Fang X, et al. Physical activity in aging: Comparison among young, aged, and nonagenarian individuals. J Appl Physiol. 2008;105(2):495–501. doi: 10.1152/japplphysiol.90450.2008 18556430 PMC2519943

[6] Pancani S, Vannetti F, Sofi F, Cecchi F, Pasquini G, Fabbri L, et al. Association between physical activity and functional and cognitive status in nonagenarians: results from the Mugello study. Int Psychogeriatrics. 2019;31(06):901–8. doi: 10.1017/S1041610218001424 30560746

[7] Pes GM, Dore MP, Errigo A, Poulain M. Analysis of physical activity among free-living nonagenarians from a Sardinian Longevous Population. J Aging Phys Act. 2018;26(2):254–8. doi: 10.1123/japa.2017-0088 28714795

[8] Bellettiere J, Carlson JA, Rosenberg D, Singhania A, Natarajan L, Berardi V, et al. Gender and age differences in hourly and daily patterns of sedentary time in older adults living in retirement communities. PLoS One. 2015;10(8):e0136161. doi: 10.1371/journal.pone.0136161 26296095 PMC4546658

[9] Kaprio J, Bollepalli S, Buchwald J, Iso-Markku P, Korhonen T, Kovanen V, et al. The Older Finnish Twin Cohort—45 years of follow-up. Twin Res Hum Genet. 2019;22(4):240–54. doi: 10.1017/thg.2019.54 31462340

[10] Brumback-Peltz C, Balasubramanian AB, Corrada MM, Kawas CH. Diagnosing dementia in the oldest-old. Maturitas. 2011;70(2):164–8. doi: 10.1016/j.maturitas.2011.07.008 21831546 PMC3171568

[11] Kawas CH, Kim RC, Sonnen JA, Bullain SS, Trieu T, Corrada MM. Multiple pathologies are common and related to dementia in the oldest-old. Neurology. 2015;85(6):535–42. 26180144 10.1212/WNL.0000000000001831 PMC4540246

[12] Waller K, Kaprio J, Kujala UM. Associations between long-term physical activity, waist circumference and weight gain: a 30-year longitudinal twin study. Int J Obes (Lond). 2008;32(2):353–61. doi: 10.1038/sj.ijo.0803692 17653065

[13] Leskinen T, Waller K, Mutikainen S, Aaltonen S, Ronkainen PH, Alen M, et al. Effects of 32-year leisure time physical activity discordance in twin pairs on health (TWINACTIVE study): aims, design and results for physical fitness. Twin Res Hum Genet. 2009;12(1):108–17. doi: 10.1375/twin.12.1.108 19210186

[14] Radloff LS. The CES-D Scale. Appl Psychol Meas. 1977;1(3):385–401.

[15] Vilagut G, Forero CG, Barbaglia G, Alonso J. Screening for depression in the general population with the Center for Epidemiologic Studies Depression (CES-D): A systematic review with meta-analysis. PLoS One. 2016;11(5):e0155431. doi: 10.1371/journal.pone.0155431 27182821 PMC4868329

[16] Beekman AT, Deeg DJ, Van Limbeek J, Braam AW, De Vries MZ, Van Tilburg W. Criterion validity of the Center for Epidemiologic Studies Depression scale (CES-D): results from a community-based sample of older subjects in The Netherlands. Psychol Med. 1997;27(1):231–5. doi: 10.1017/s0033291796003510 9122304

[17] Gatz M, Johansson B, Pedersen N, Berg S, Reynolds C. A cross-national self-report measure of depressive symptomatology. Int psychogeriatrics. 1993;5(2):147–56. doi: 10.1017/s1041610293001486 8292768

[18] Lewinsohn PM, Seeley JR, Roberts RE, Allen NB. Center for Epidemiologic Studies Depression Scale (CES-D) as a screening instrument for depression among community-residing older adults. Psychol Aging. 1997;12(2):277–87. doi: 10.1037//0882-7974.12.2.277 9189988

[19] Scott B, Melin L. Psychometric properties and standardised data for questionnaires measuring negative affect, dispositional style and daily hassles. A nation-wide sample. Scand J Psychol. 1998;39(4):301–7.

[20] Gatz M, Reynolds C, Nikolic J, Lowe B, Karel M, Pedersen N. An empirical test of telephone screening to identify potential dementia cases. Int Psychogeriatrics. 1995;7(3):429–38. doi: 10.1017/s1041610295002171 8821350

[21] Järvenpää T, Rinne JO, Räihä I, Koskenvuo M, Löppönen M, Hinkka S, et al. Characteristics of two telephone screens for cognitive impairment. Dement Geriatr Cogn Disord. 2002;13(3):149–55. doi: 10.1159/000048646 11893836

[22] Laitala VS, Kaprio J, Koskenvuo M, Räihä I, Rinne JO, Silventoinen K. Coffee drinking in middle age is not associated with cognitive performance in old age. Am J Clin Nutr. 2009;90(3):640–6. doi: 10.3945/ajcn.2009.27660 19587088

[23] Jason Brandt, Miriam Spencer, Marshal Folstein. The telephone interview for cognitive status. Neuropsychiatry, Neuropsychol Behav Neurol. 1988;1(2):111–7.

[24] Gatz M, Reynolds CA, John R, Johansson B, Mortimer JA, Pedersen NL. Telephone screening to identify potential dementia cases in a population-based sample of older adults. Int psychogeriatrics. 2002;14(3):273–89. 10.1017/s1041610202008475 12475088

[25] Lindgren N, Rinne JO, Palviainen T, Kaprio J, Vuoksimaa E. Prevalence and correlates of dementia and mild cognitive impairment classified with different versions of the modified Telephone Interview for Cognitive Status (TICS‐m). Int J Geriatr Psychiatry. 2019;34(12):1883–91. doi: 10.1002/gps.5205 31469194

[26] Vasankari V, Halonen J, Husu P, Vähä-Ypyä H, Tokola K, Suni J, et al. Personalised eHealth intervention to increase physical activity and reduce sedentary behaviour in rehabilitation after cardiac operations: study protocol for the PACO randomised controlled trial (NCT03470246). BMJ Open Sport Exerc Med. 2019;5(1):e000539. doi: 10.1136/bmjsem-2019-000539 31354960 PMC6615853

[27] Husu P, Tokola K, Vähä-Ypyä H, Sievänen H, Suni J, Heinonen OJ, et al. Physical activity, sedentary behavior, and time in bed among Finnish adults measured 24/7 by triaxial accelerometry. J Meas Phys Behav. 2021;4(2):163–73.

[28] Vähä-Ypyä H, Vasankari T, Husu P, Suni J, Sievänen H. A universal, accurate intensity-based classification of different physical activities using raw data of accelerometer. Clin Physiol Funct Imaging. 2015;35(1):64–70. doi: 10.1111/cpf.12127 24393233

[29] Vähä-Ypyä H, Vasankari T, Husu P, Mänttäri A, Vuorimaa T, Suni J, et al. Validation of cut-points for evaluating the intensity of physical activity with accelerometry-based mean amplitude deviation (MAD). PLoS One. 2015;10(8):e0134813. doi: 10.1371/journal.pone.0134813 26292225 PMC4546343

[30] Vähä-Ypyä H, Husu P, Suni J, Vasankari T, Sievänen H. Reliable recognition of lying, sitting, and standing with a hip-worn accelerometer. Scand J Med Sci Sports. 2018;28(3):1092–102. doi: 10.1111/sms.13017 29144567

[31] Winter DA, Patla AE, Frank JS, Walt SE. Biomechanical walking pattern changes in the fit and healthy elderly. Phys Ther. 1990;70(6):340–7. doi: 10.1093/ptj/70.6.340 2345777

[32] Matthews CE, Hagströmer M, Pober DM, Bowles HR. Best practices for using physical activity monitors in population-based research. Med Sci Sports Exerc. 2012;44(1 Suppl 1):S68–76. doi: 10.1249/MSS.0b013e3182399e5b 22157777 PMC3543867

[33] Tudor-Locke C, Craig CL, Brown WJ, Clemes SA, De Cocker K, Giles-Corti B, et al. How many steps/day are enough? for adults. Int J Behav Nutr Phys Act. 2011;8(1):79. doi: 10.1186/1479-5868-8-79 21798015 PMC3197470

[34] Lee IM, Shiroma EJ, Kamada M, Bassett DR, Matthews CE, Buring JE. Association of step volume and intensity with all-cause mortality in older women. JAMA Intern Med. 2019;179(8):1105. doi: 10.1001/jamainternmed.2019.0899 31141585 PMC6547157

[35] Paluch AE, Bajpai S, Bassett DR, Carnethon MR, Ekelund U, Evenson KR, et al. Daily steps and all-cause mortality: a meta-analysis of 15 international cohorts. Lancet Public Heal. 2022;7(3):e219–28. doi: 10.1016/S2468-2667(21)00302-9 35247352 PMC9289978

[36] Straczkiewicz M, Glynn NW, Harezlak J. On placement, location and orientation of wrist-worn tri-axial accelerometers during free-living measurements. Sensors. 2019;19(9):2095. doi: 10.3390/s19092095 31064100 PMC6538999

[37] Frisard MI, Fabre JM, Russell RD, King CM, DeLany JP, Wood RH, et al. Physical activity level and physical functionality in nonagenarians compared to individuals aged 60–74 years. Journals Gerontol Ser A Biol Sci Med Sci. 2007;62(7):783–8. doi: 10.1093/gerona/62.7.783 17634327 PMC2724866

[38] dos Santos CES, d’Orsi E, Rech CR. Association between different cutoff points for objectively measured moderate-to-vigorous physical activity and cardiometabolic markers in older adults. Arch Gerontol Geriatr. 2020;91:104238. doi: 10.1016/j.archger.2020.104238 32861953

[39] Vähä-Ypyä H, Sievänen H, Husu P, Tokola K, Vasankari T. Intensity paradox–low-fit people are physically most active in terms of their fitness. Sensors. 2021;21(6):2063. doi: 10.3390/s21062063 33804220 PMC8002087

[40] Kujala UM, Pietilä J, Myllymäki T, Mutikainen S, Föhr T, Korhonen I, et al. Physical activity: absolute intensity vs. relative-to-fitness-level volumes. Med Sci Sport Exerc. 2017;49(3):474–81. 10.1249/MSS.0000000000001134 27875497

[41] Domingos C, Correia Santos N, Pêgo JM. Association between self-reported and accelerometer-based estimates of physical activity in portuguese older adults. Sensors. 2021;21(7):2258. doi: 10.3390/s21072258 33804834 PMC8038119

[42] Cerin E, Cain KL, Oyeyemi AL, Owen N, Conway TL, Cochrane T, et al. Correlates of agreement between accelerometry and self-reported physical activity. Med Sci Sport Exerc. 2016;48(6):1075–84. doi: 10.1249/MSS.0000000000000870 26784274 PMC4868646

[43] Lim K, Taylor L. Factors associated with physical activity among older people–a population-based study. Prev Med (Baltim). 2005;40(1):33–40. doi: 10.1016/j.ypmed.2004.04.046 15530578

[44] Sumic A, Michael YL, Carlson NE, Howieson DB, Kaye JA. Physical activity and the risk of dementia in oldest old. J Aging Health. 2007;19(2). doi: 10.1177/0898264307299299 17413134 PMC3110722

[45] Hall A, Pekkala T, Polvikoski T, Van Gils M, Kivipelto M, Lötjönen J, et al. Prediction models for dementia and neuropathology in the oldest old: The Vantaa 85+ cohort study. Alzheimer’s Res Ther. 2019;11(1). doi: 10.1186/s13195-018-0450-3 30670070 PMC6343349

[46] Ruiz JR, Gil-Bea F, Bustamante-Ara N, Rodríguez-Romo G, Fiuza-Luces C, Serra-Rexach JA, et al. Resistance training does not affect cognition or related serum biomarkers in nonagenarians: a randomized controlled trial. Int J Sports Med. 2015;36(1):54–60. 25329433 10.1055/s-0034-1375693

[47] Miller KJ, Suárez-Iglesias D, Varela S, Rodríguez D, Ayán C. Exercise for nonagenarians: a systematic review. J Geriatr Phys Ther. 2020;43(4):208–18. doi: 10.1519/JPT.0000000000000245 31569172

[48] Idland G, Sylliaas H, Mengshoel AM, Pettersen R, Bergland A. Progressive resistance training for community-dwelling women aged 90 or older: a single-subject experimental design. Disabil Rehabil. 2014;36(15):12401248. doi: 10.3109/09638288.2013.837969 24093596

[49] Husu P, Suni J, Vähä-Ypyä H, Sievänen H, Tokola K, Valkeinen H, et al. Objectively measured sedentary behavior and physical activity in a sample of Finnish adults: a cross-sectional study. BMC Public Health. 2016;16(1):920. doi: 10.1186/s12889-016-3591-y 27586887 PMC5009485

[50] Barnes JC, Boutwell BB. A Demonstration of the generalizability of twin-based research on antisocial behavior. Behav Genet. 2013;43(2):120–31. doi: 10.1007/s10519-012-9580-8 23274656 PMC3683969

[51] Evans DM, Martin NG. The validity of twin studies. GeneScreen. 2000;1(2):77–9.

[52] Skytthe A, Harris JR, Czene K, Mucci L, Adami HO, Christensen K, et al. Cancer incidence and mortality in 260,000 Nordic twins with 30,000 prospective cancers. Twin Res Hum Genet. 2019;22(2):99–107. doi: 10.1017/thg.2019.10 31020942

