# Supplementary material for: Accelerometer-measured physical activity and sedentary behavior in nonagenarians: Associations with self-reported physical activity, anthropometric, sociodemographic, health and cognitive characteristics
Source: PLoS One. 2023 Dec 6;18(12):e0294817. doi: 10.1371/journal.pone.0294817 (PMC10699641; doi:10.1371/journal.pone.0294817)
Supplement: S1 File — (DOCX) [file pone.0294817.s001.docx]

**S1 Material. Physical activity questions used in the NONAGINTA – Memory and Health in 90-year-olds – study.**

Here are five alternatives to choose from when deciding on the amount of physical activity you get. How do you describe the amount of your year-round physical activity?

1) practically none

2) a little

3) a moderate amount

4) quite a lot

5) a great deal

How often do you exercise or be physically active in a month?

1) less than once a month

2) 1–2 times a month

3) 3–5 times a month

4) 6–10 times a month

5) 11–19 times a month

6) more than 20 times a month

How long does one session of the physical activity last on average?

1) less than 15 min

2) 15 min to less than half an hour

3) half an hour to less than one hour

4) one hour to under two hours

5) two hours or more

Is your physical activity about as tiring on average as:

1) walking

2) alternatively walking and jogging

3) jogging (light run)

4) running
