# Supplementary material for: Accelerometer-measured physical activity and sedentary behavior in nonagenarians: Associations with self-reported physical activity, anthropometric, sociodemographic, health and cognitive characteristics
Source: PLoS One. 2023 Dec 6;18(12):e0294817. doi: 10.1371/journal.pone.0294817 (PMC10699641; doi:10.1371/journal.pone.0294817)
Supplement: S1 Table — (DOCX) [file pone.0294817.s002.docx]

**S1 Table. Demographics and cognitive, health, and physical activity characteristics in nonagenarian men (n=19) and women (n=19).**

| **Characteristics** | **Men** | **Women** |  |
| --- | --- | --- | --- |
|  | Mean (*SD*) | Mean (*SD*) | p-value |
| **Age** (years) | 91.0 (1.3) | 91.5 (1.9) | 0.42 |
| **Height** (cm) | 172.7 (5.5) | 155.8 (4.9) | <0.001 |
| **Weight** (kg) | 72.7 (9.2) | 62.8 (10.0) | 0.01 |
| **BMI** (kg/m^2^) | 24.4 (2.9) | 25.9 (4.1) | 0.21 |
| **Education** |  |  | 0.91 |
| <Compulsory education | 0 | 0 |  |
| Compulsory education | 7 | 8 |  |
| Compulsory education and ≥ 1-year  vocational training | 5 | 1 |  |
| Lower secondary education | 0 | 1 |  |
| Lower secondary education and ≥ 1-year  vocational training | 1 | 3 |  |
| Upper secondary education | 0 | 0 |  |
| Upper secondary education and ≥ 1-year vocational training | 0 | 1 |  |
| Tertiary education (university or polytechnic college) | 4 | 4 |  |
| Other education | 2 | 1 |  |
| **Subjective health status** |  |  | 0.19 |
| Very poor | 0 | 0 |  |
| Poor | 1 | 3 |  |
| Fair | 12 | 13 |  |
| Good | 6 | 3 |  |
| **Dizziness/poor balance** |  |  | 0.80 |
| Never or hardly ever | 10 | 11 |  |
| Sometimes | 8 | 7 |  |
| Often | 1 | 1 |  |
| **Fear of falling** |  |  | 0.33 |
| Never | 9 | 6 |  |
| Occasionally | 7 | 10 |  |
| Often | 3 | 1 |  |
| Constantly | 0 | 2 |  |
| **Words recalled in three trials** | 12.2 (3.6)^a^ | 12.6 (3.9) | 0.71 |
| **Delayed word list recall** | 1.8 (1.7)^a^ | 2.6 (2.4) | 0.28 |
| **Semantic fluency** | 17.4 (4.4)^a^ | 16.9 (3.5) | 0.67 |
| **TELE category** |  |  | 0.56 |
| Cognitive impairment | 4^a^ | 2 |  |
| Mild impairment | 6^a^ | 8 |  |
| Cognitively healthy | 8^a^ | 9 |  |
| **Depressive symptoms*** | 13.2 (4.5)^a^ | 15.3 (7.1)^a^ | 0.27 |
| **Clinically significant depressive symptoms** |  |  | 0.30 |
| Yes | 3 | 1 |  |
| No | 16 | 18 |  |
| **Amount of physical activity** |  |  | 0.27 |
| None/very little | 0 | 4 |  |
| Little | 4 | 2 |  |
| Moderate | 10 | 9 |  |
| Quite a lot | 5 | 4 |  |
| A great deal | 0 | 0 |  |
| **Physical activity frequency** |  |  | 0.03 |
| Less than once a month | 0 | 1^b^ |  |
| 1–2 times a month | 0 | 0^b^ |  |
| 3–5 times a month | 3 | 3^b^ |  |
| 6–10 times a month | 1 | 6^b^ |  |
| 11–19 times a month | 4 | 3^b^ |  |
| More than 20 times a month | 11 | 3^b^ |  |
| **Physical activity duration** |  |  | 0.85 |
| Less than 15 min | 1 | 1^b^ |  |
| 15 min to less than 30 min | 4 | 3^b^ |  |
| 30 min to less than 1 hour | 9 | 7^b^ |  |
| 1 hour to under 2 hours | 5 | 5^b^ |  |
| 2 hours or more | 0 | 0^b^ |  |
| **Physical activity intensity** |  |  | 0.20 |
| Walking | 14 | 15^c^ |  |
| Alternatively walking and jogging | 4 | 2^c^ |  |
| Jogging | 1 | 0^c^ |  |
| Running | 0 | 0^c^ |  |
| **MET hours/day** | 2.3 (1.7) | 1.5 (1.3)^b^ | 0.10 |

*Notes*: SD=standard deviation; BMI=body mass index; TELE=Telephone assessment for dementia; MET=metabolic equivalent.

* Sum score ranging 0–60, higher values indicate depressive symptoms; ^a^=the number of participants is 18; ^b^= the number of participants is 16; ^c^=the number of participants is 17.
